# Association of Cumulative Exposure to Metabolic Score for Visceral Fat With the Risk of Cardiovascular Disease and All‐Cause Mortality: A Prospective Cohort Study

**DOI:** 10.1002/jcsm.13702

**Published:** 2025-02-11

**Authors:** Qian Liu, Haozhe Cui, Fei Si, Yuntao Wu, Jing Yu

**Affiliations:** ^1^ Department of Cardiology Lanzhou University Second Hospital Lanzhou China; ^2^ The Second Clinical Medical School Lanzhou University Lanzhou China; ^3^ School of Medicine Nankai University Tianjin China; ^4^ Department of Cardiology Kailuan General Hospital Tangshan China

**Keywords:** cardiovascular disease, cumulative, metabolic score for visceral fat, prospective cohort study

## Abstract

**Background:**

Previous studies have demonstrated that metabolic score for visceral fat (METS‐VF), a novel surrogate indicator assessing visceral fat, was associated with the risk of hypertension, diabetes mellitus, cardiovascular disease (CVD) and mortality, predicting the risks based on a single METS‐VF measurement can increase limitations of the study. Few studies have investigated the association between cumulative exposure to METS‐VF and risk of CVD and all‐cause mortality. We aimed to examine the association of cumulative METS‐VF with risk for CVD and all‐cause mortality.

**Methods:**

All participants in the study were from the Kailuan Study, which is a large, prospective cohort study, and began in 2006 years. Cumulative METS‐VF was calculated by data from 2006 survey to 2010 survey and defined as the mean METS‐VF for each pair of consecutive surveys multiplied by the time intervals between these two consecutive surveys. The optimal cut‐off value for time‐averaged cumulative METS‐VF associated with CVD was determined using a survival‐time method to calculate maximally selected rank statistics and was used to assess exposure of high METS‐VF. A multivariate Cox proportional hazards regression model was used to assess the risk of CVD and all‐cause mortality during 2010–2022 years (hazard ratio [HR] and 95% confidence interval [95% CI]).

**Results:**

We included 41 756 participants (mean age, [52.72 ± 11.64] years, 78.53% males and 21.47% females). All participants were divided into four groups: Q1 (reference group), Q2, Q3 and Q4 according to the quartiles of cumulative METS‐VF, and exposure duration of high METS‐VF was quantified as 0, 2, 4, and 6 years. During the median follow‐up of 12.01 years, 4008 (9.60%) CVD events and 3944 all‐cause mortality events occurred. After adjusting for potential covariates, compared to participants in Q1 group, the HRs of incident CVD and all‐cause mortality were 1.55 (95% CI, 1.38–1.74) and 1.59 (95% CI, 1.40–1.81) for those in Q2 group, 2.13 (95% CI, 1.91–2.38) and 2.67 (95% CI 2.37–3.01) for those in Q3 group, 2.78 (95% CI, 2.49–3.17) and 4.90 (95% CI 4.36–5.50) for those in Q4 group. The HRs for CVD and all‐cause mortality were increased with exposure duration of high METS‐VF increasing. The result of ROC curve analysis showed that cumulative METS‐VF had the highest predictive for CVD among 4 indexes including cumulative METS‐VF, cumulative waist circumference, cumulative body mass index and cumulative WHtR.

**Conclusions:**

The high cumulative METS‐VF was associated with an increased risk of CVD and mortality, and this association was stronger as exposure to high METS‐VF was prolonged, emphasizing the importance of striving to control the METS‐VF.

**Abbreviations**
95% CI: 95% confidence interval
ALT: alanine aminotransferase
AUC: area under the curve
BMI: body mass index
CVD: cardiovascular disease
DBP: diastolic blood pressure
eGFR: estimated glomerular filtration rate
FBG: fast blood glucose
HDL‐C: high‐density lipoprotein cholesterol
HR: hazard ratio
hs‐CRP: high‐sensitive C‐reactive protein
ICD: International Classification of Diseases
LDL‐C: low‐density lipoprotein cholesterol
METS‐IR: metabolic score for insulin resistance
METS‐VF: metabolic score for visceral fat
Q: quartile
ROC: receiver operating characteristic
SBP: systolic blood pressure
SD: standard
TG: triglyceride
WHtR: waist‐to‐height ratio

## 1 Introduction

Despite a decrease in age‐standardized cardiovascular mortality rates, cardiovascular disease (CVD) remains the leading cause of death worldwide due to the ageing global population and preventable metabolic, behavioural or environmental risk factors. Moreover, China has the highest mortality rates worldwide [1]. The incidence and number of deaths from CVD have shown an increasing trend among Chinese residents, primarily due to the prevalence of unhealthy lifestyles and the aging population [2]. Therefore, CVD is a major public health challenge in China and worldwide. Primary prevention is one of the most effective and the most fundamental measures to prevent CVD. In a simplified view, early identification of future disease risks using simple markers can prevent and improve existent risk factors, thereby promoting CVD prevention.

Obesity refers to excess body fat and abnormal body fat distribution and has been confirmed as an independent risk factor for many chronic diseases such as CVD [1, 3]. Among deaths due to metabolic diseases such as hypertension, diabetes mellitus, hyperlipidaemia, obesity and non‐alcoholic fatty liver disease, the absolute burden of obesity is highest and presents a steady upward trend [4]. Generally, obesity is mainly defined by body mass index (BMI). However, BMI, as well as other visceral fat indicators such as waist circumference, waist‐to‐hip ratio and waist‐to‐height ratio (WHtR), cannot distinguish between visceral and subcutaneous fat [5]. Visceral fat is a marker of CVD that is independent of BMI and is associated with increased risk of cardiometabolic risk factors [6, 7]. Currently, the metabolic score for visceral fat (METS‐VF), a novel surrogate indicator calculated based on the metabolic score for insulin resistance (METS‐IR), WHtR, age and sex, has been shown to effectively assess visceral fat. METS‐VF has better performance compared with other visceral fat indicators, magnetic resonance imaging (a gold standard to assess visceral fat), and other imaging techniques, such as computerized tomography, dual x‐ray absorptiometry and bioelectrical impedance analysis. In addition, METS‐VF has been applied to assess cardiometabolic risk in clinical and epidemiological research and has been proposed to be associated with increased risk of hypertension, diabetes mellitus, CVD and mortality [7, 8]. METS‐VF is influenced by biochemical and environmental factors; previous studies were mainly based on a single measurement, which could not reflect the longitudinal changes and cumulative burden related to elevated METS‐VF. In contrast, several prospective cohort studies have utilized the cumulative exposure and exposure duration, which could reflect the intensity and fluctuations of METS‐VF over an extended duration [9, 10]. However, studies on the association between cumulative METS‐VF and the risk of CVD and all‐cause mortality are lacking.

Therefore, this study employed a large cohort prospective cohort design to investigate the association between the cumulative burden of METS‐VF and high METS‐VF and the risk of CVD and all‐cause mortality.

## 2 Methods

### 2.1 Study Participants

Kailuan Study is an ongoing prospective cohort study based on the Kailuan community in Tangshan City, China. All participants enrolled in the Kailuan Study are employees and retirees of the Kailuan group, which is famous for its coal industry. Details of study design and implementation have been described previously [11, 12]. Briefly, from June 2006 to October 2006 (abbreviated as 2006 survey), 101 510 participants were observed in biennial questionnaire, clinical test and laboratory test at the Kailuan General Hospital and their affiliated 10 hospitals.

**FIGURE 1:**
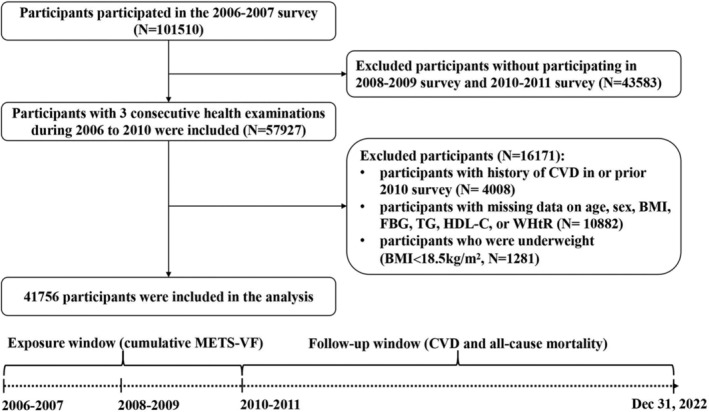
Flowchart of the participants in the current analysis. BMI, body mass index; CVD, cardiovascular disease; FBG, fasting blood glucose; HDL‐C, high‐density lipoprotein cholesterol; METS‐VF, metabolic score for visceral fat; TG, triglyceride; WHtR, waist‐to‐height ratio.

In the current study, cumulative METS‐VF was calculated by data from 2006 survey to 2010 survey to observe the incident risk of CVD and all‐cause mortality during 2010–2022. We initially included 101 510 participants. Next, we excluded 43 583 participants who did not attend 2008 survey or 2010 survey, 4008 participants with history of CVD in 2010 survey, 10 882 participants with missing data on age, sex, BMI, fast blood glucose (FBG), triglyceride (TG), low‐density lipoprotein cholesterol (LDL‐C) or WHtR and 1281 participants who were underweight (BMI < 18.5 kg/m^2^), 41 756 participants were eligible according to the inclusion and exclusion criteria (Figure 1). In addition to WHtR levels and the proportion of current smokers, the characteristics of included and excluded participants in 2006 were statistically different because of inclusion of participants with CVD history in excluded participants (Table S1).

The study complied with the declaration of Helsinki and was approved by the Ethics Committee of the Kailuan General Hospital. All participants signed written informed consent forms.

### 2.2 Data Collection

Information on demographic characteristics (age, sex and identification number), lifestyles (smoke, drink and physical activity), medication use (antihypertensive medications, hypoglycaemic medications and lipid‐lowering medications), medical history (a history of CVD and cancer) was collected via face‐to‐face questionnaires by specialists with professional training, as described elsewhere [12]. Blood samples were collected from participants fasting for at least 8 h and analysed for biochemical index such as TG, LDL‐C, high‐density lipoprotein cholesterol (HDL‐C), FBG, creatinine, alanine aminotransferase (ALT) and high‐sensitive C‐reactive protein (hs‐CRP) in a Hitachi 7600 auto‐analyser (Hitachi, Tokyo, Japan) [13]. Brachial blood pressure, height, weight and waist circumference were measure using standard procedures. BMI (kg/m^2^) was calculated by weight (kg) divided by the square of height (m^2^) and categorized as less than 24 kg/m^2^ and 24 kg/m^2^ or higher [14]. WHtR was calculated by waist circumference (cm) divided by height (cm). Hypertension was defined by systolic blood pressure (SBP) ≥ 140 mmHg and/or diastolic blood pressure (DBP) ≥ 90 mmHg (1 mmHg = 0.133 kPa), or a history of hypertension, or the use of antihypertensive medications. Diabetes mellitus was defined by FBG ≥ 7 mmol/L, or a history of diabetes mellitus or the use of hypoglycaemic medications. Dyslipidaemia was defined as total cholesterol ≥ 6.2 mmol/L, TG ≥ 2.3 mmol/L, LDL‐C ≥ 4.1 mmol/L, HDL‐C < 1.04 mmol/L or the use of lipid‐lowering medications. Hs‐CRP was categorized as less than 3 mg/dL and 3 mg/dL or higher. The estimated glomerular filtration rate (eGFR) was calculated according to the Chronic Kidney Disease Epidemiology Collaboration formula [15]. Current smokers and drinkers were classified as yes or no. Active physical activity was defined as exercising at least 4 times per week, with at least 20 min. Educational status was categorized as less than high school and high school or above.

### 2.3 Assessment of Cumulative METS‐VF and High METS‐VF

METS‐IR and METS‐VF was calculated as follows: [7, 16]

$$\text{METS}-\mathrm{IR}=\left[\ln\left(\left(2\times \mathrm{FBG}\right)+\mathrm{TG}\right)\times \mathrm{BMI}\right]/\left[\ln\;\left(\mathrm{HDL}-C\right)\right]$$

$$\begin{array}{c} \text{METS}-\mathrm{VF}=4.466+0.011\times \left[{\left(\ln\;\left(\text{METS}-\mathrm{IR}\right)\right)}^{3}\right]+3.239\times \\ \;\left[{\left(\ln\;\left(\text{WHtR}\right)\right)}^{3}\right]+0.319\times \mathrm{sex}+0.594\times \left[\ln\;\left(\mathrm{age}\right)\right] \end{array}$$

where sex was a binary variable (male = 1, female = 0) and age was coded in years.

Cumulative METS‐VF was defined as the mean METS‐VF for each pair of consecutive surveys multiplied by the time intervals between these two consecutive surveys [17, 18] and calculated as follows:

$$\begin{array}{c} \begin{array}{c} \begin{array}{c} \text{Cumulative METS}-\mathrm{VF}=\\ \;\left[\left(\text{METS}-{\mathrm{VF}}_{2006}+\text{METS}-{\mathrm{VF}}_{2008}\right)/2\times {\text{time}}_{2006–2008}\right]\\ \;+\left[\left(\text{METS}-{\mathrm{VF}}_{2008}+\text{METS}-{\mathrm{VF}}_{2010}\right)/2\times {\text{time}}_{2008–2010}\right] \end{array} \end{array} \end{array}$$

where METS‐VF_2006_, METS‐VF_2008_, METS‐VF_2010_ indicated METS‐VF at the 2006 survey, 2008 survey and 2010 survey, time_2006–2008_ and time_2008–2010_ indicated time intervals between consecutive surveys. According to the quartiles (Q) of cumulative METS‐VF, all participants were divided into four groups: Q1 (< 23.86, reference group), Q2 (23.86–25.90), Q3 (25.91–28.48) and Q4 (≥ 28.49).

The cut‐off value for the definition of high METS‐VF exposure was mentioned statistical analysis, and exposure duration of high METS‐VF was defined as the number of visits with high METS‐VF at three surveys, quantified as 0 years (without high METS‐VF, reference group), 2 years (had high METS‐VF once), 4 years (had high METS‐VF twice) and 6 years (had high METS‐VF at three surveys).

### 2.4 Cardiovascular Disease and All‐Cause Mortality Ascertainment

The follow‐up time was calculated as the time from baseline (2010 survey) to the date of CVD incident, the date of all‐cause mortality or 31 December 2022, whichever came first. In addition to collecting a history of CVD in annual survey, the onset of CVD, encompassed myocardial infarction (I21), stroke (I60, I61 and I63) and heart failure (I50), was collected from Tangshan medical insurance system and the social security system of the Kailuan Group annually, was diagnosed by trained medical staff and was coded according to the International Classification of Diseases, 10th revision (ICD‐10). According to World Health Organization criteria, stroke was defined based on signs and symptoms of neurological deficit, and in imaging tests such as head computerized tomography or magnetic resonance imaging scans, acute myocardial infarction was defined based on clinical symptoms, electrocardiogram and dynamic changes in cardiac enzymes [19, 20]. According to the China guidelines for the diagnosis and treatment of heart failure 2018 [21], heart failure was diagnosed based on the following criteria (containing at least terms 1 and 2): (1) symptoms of heart failure (including dyspnoea, fatigue and fluid retention), and the diagnosis was in New York Heart Association class II–IV or Killip class II‐IV at the times of discharge; (2) the modified Simpson method of two‐dimensional and Doppler echocardiographic showed a left ventricular ejection fraction ≤ 50%; and (3) elevated plasma N‐terminal B‐type natriuretic peptide levels. Data on all‐cause death were collected via death certificate from provincial population statistics office [22].

### 2.5 Statistical Analyses

The optimal cut‐off value for time‐averaged cumulative METS‐VF associated with CVD incident was determined using a survival‐time method to calculate maximally selected rank statistics with maxstat package and relevant packages, which can be found in supplement material and previous study [23].

Depending on distribution status, variables were expressed as mean ± standard (SD) or interquartile range and were compared using variance analysis and Mann–Whitney *U* test between groups. Categoric variables were expressed as number and percentage and compared using chi‐squared test. Because the proportion of missing values of covariates was below 5%, we used the chain equations to impute missing values on covariates.

The cumulative incidence of CVD and all‐cause mortality were calculated using the Kaplan–Meier method, and the differences were compared using log‐rank test. Incidence rates per 1000 person‐years were calculated for CVD and all‐cause mortality. The proportional hazards assumption was tested and satisfied by the Schoenfeld residual method, so a multivariate Cox proportional hazards regression model was used to assess the incidence of CVD and all‐cause mortality (hazard ratio [HR] and 95% confidence interval [95% CI]). All models adjusted for LDL‐C, ALT, hs‐CRP categories, BMI categories, hypertension, diabetes mellitus, dyslipidaemia, drinking status, smoking status, physical exercise and educational status. The *p* values for trend were calculated by treating categorical variables (quartiles of cumulative METS‐VF and exposure duration of high METS‐VF) as continuous variables. We also calculated the risk of CVD and all‐cause mortality for per SD increment in cumulative METS‐VF (SD = 3.44). The dose–response associations between continuous cumulative METS‐VF and the risk of CVD/all‐cause mortality were assessed using restricted cubic splines with three knots (25th, 50th and 75th percentiles).

Receiver operating characteristic (ROC) curves were established; predictive performances of cumulative METS‐VF, cumulative BMI, cumulative waist circumference and cumulative WHtR for CVD and all‐cause mortality were compared by the area under the curve (AUC). The optimal cut‐off values were calculated using the Youden index.

To test results stability, we conducted several subgroup analyses and sensitivity analyses: (1) We performed several subgroup analyses for the association of per SD increment in cumulative METS‐VF with the risk of cardiovascular disease and all‐cause mortality by age (< 60 years and ≥ 60 years), sex (males and females), BMI (< 24 kg/m^2^ and ≥ 24 kg/m^2^), WHtR (< 0.5 and ≥ 0.5), hypertension (yes and no), diabetes mellitus (yes and no), dyslipidaemia (yes and no), and hs‐CRP (< 3 mg/dL and ≥ 3 mg/dL). The multiplicative interaction was calculated via Z test; we also compared the ratio of HR (95% CI) between two groups. (2) We further analysed the associations of METS‐VF with the risk of CVD subtypes, including myocardial infarction, stroke and heart failure. (3) We performed several sensitivity analyses by excluding participants with incident CVD within 2 years to reduce the possibility of reverse causality; excluding participants with eGFR lower than 60 mL/min per 1.73 m^2^; excluding participants with use of antihypertensive medications, hypoglycaemic medications or lipid‐lowering medications; or excluding participants with history of cancer. (4) Considering competing relationship between CVD and death, the analysis for the association of cumulative METS‐VF with the risk of CVD was conducted using competing risk model to remove estimation biases from competing events.

All statistical analyses were conducted using SAS Version 9.4 (SAS Institute, Cary, NC) and R Version 4.3.1; two‐sided *p* values <0.05 were considered statistically significant.

## 3 Results

### 3.1 Baseline Characteristics

The comparisons of all participants according to the quartiles of cumulative METS‐VF are presented in Table 1. Of the 41 756 participants, the mean age was (52.72 ± 11.64) years, 78.53% were males, and 21.47% females. Compared with the participants in Q1 group, those in the higher quartiles were likely to be older and less educated; had higher levels of BMI, WHtR, HDL‐C and hs‐CRP; and had higher prevalence of hypertension, diabetes mellitus, dyslipidaemia and higher usage of antihypertensive medications, hypoglycaemic medications and lipid‐lowering medications.

**TABLE 1:** Baseline characteristics of participants according to quartiles of cumulative METS‐VF.

		Quartiles of METS‐VF				
Characteristics	Overall	Q1 (< 23.86)	Q2 (23.86–25.90)	Q3 (25.91–28.48)	Q4 (≥ 28.49)	*p*
Number of participants	41 756	10 422	10 447	10 435	10 452	
Age, years	52.72 ± 11.64	44.87 ± 9.51	49.32 ± 9.27	55.08 ± 10.09	61.59 ± 10.29	< 0.01
Male, *n* (%)	32 792 (78.53)	7025 (67.41)	8679 (83.08)	8329 (79.82)	8759 (83.80)	< 0.01
BMI, kg/m^2^	25.29 ± 3.24	23.41 ± 2.69	25.21 ± 2.90	25.93 ± 3.21	26.61 ± 3.20	< 0.01
WHtR	0.53 ± 0.06	0.49 ± 0.05	0.53 ± 0.05	0.54 ± 0.06	0.56 ± 0.06	< 0.01
LDL‐C, mmol/L	2.61 ± 0.82	2.47 ± 0.72	2.61 ± 0.77	2.63 ± 0.89	2.71 ± 0.89	< 0.01
ALT, IU/L	18.00 (13.00, 24.00)	16.00 (11.80, 22.00)	18.00 (13.00, 24.00)	18.00 (13.00, 24.00)	18.00 (13.00, 24.00)	< 0.01
Hs‐CRP, mg/dL	1.08 (0.49, 2.56)	0.81 (0.30, 2.00)	1.00 (0.40, 2.41)	1.20 (0.50, 2.71)	1.36 (0.70, 2.92)	< 0.01
eGFR, mL/min/1.73m^2^	89.04 ± 19.93	94.32 ± 20.34	91.59 ± 19.83	87.52 ± 19.64	82.76 ± 17.88	< 0.01
Current smokers, *n* (%)	14 456 (34.62)	3743 (35.91)	4374 (41.87)	3500 (33.54)	2839 (27.16)	< 0.01
Current drinkers, *n* (%)	14 617 (35.01)	3674 (35.25)	4300 (41.16)	3522 (33.75)	3121 (29.86)	< 0.01
Active physical activity, *n* (%)	5609 (13.43)	1051 (10.08)	1164 (11.14)	1475 (14.14)	1919 (18.36)	< 0.01
High school or above, *n* (%)	8047 (19.27)	2613 (25.07)	1937 (18.54)	1779 (17.05)	1718 (16.44)	< 0.01
Hypertension, *n* (%)	19 970 (47.83)	3032 (29.09)	4710 (45.08)	5705 (54.67)	6523 (62.41)	< 0.01
Diabetes mellitus, *n* (%)	4664 (11.17)	519 (4.98)	949 (9.08)	1359 (13.02)	1837 (17.58)	< 0.01
Dyslipidaemia, n (%)	12 828 (30.72)	2156 (20.69)	3298 (31.57)	3564 (34.15)	3810 (36.45)	< 0.01
Antihypertensive medications, *n* (%)	6786 (16.25)	907 (8.70)	1702 (16.29)	1998 (19.15)	2179 (20.85)	< 0.01
Hypoglycaemic medications, *n* (%)	1563 (3.74)	141 (1.35)	253 (2.42)	474 (4.54)	695 (6.65)	< 0.01
Lipid‐lowering medications, *n* (%)	359 (0.86)	64 (0.61)	81 (0.78)	125 (1.20)	89 (0.85)	< 0.01

Abbreviations: ALT, alanine aminotransferase; eGFR, estimated glomerular filtration rate; Hs‐CRP, high‐sensitive C‐reactive protein; LDL‐C, low‐density lipoprotein cholesterol; METS‐VF, metabolic score for visceral fat; Q, quartile; BMI, body mass index; WHtR, waist‐to‐height ratio.

### 3.2 Cumulative Exposure to METS‐VF and Risk of CVD and All‐Cause Mortality

**FIGURE 2:**
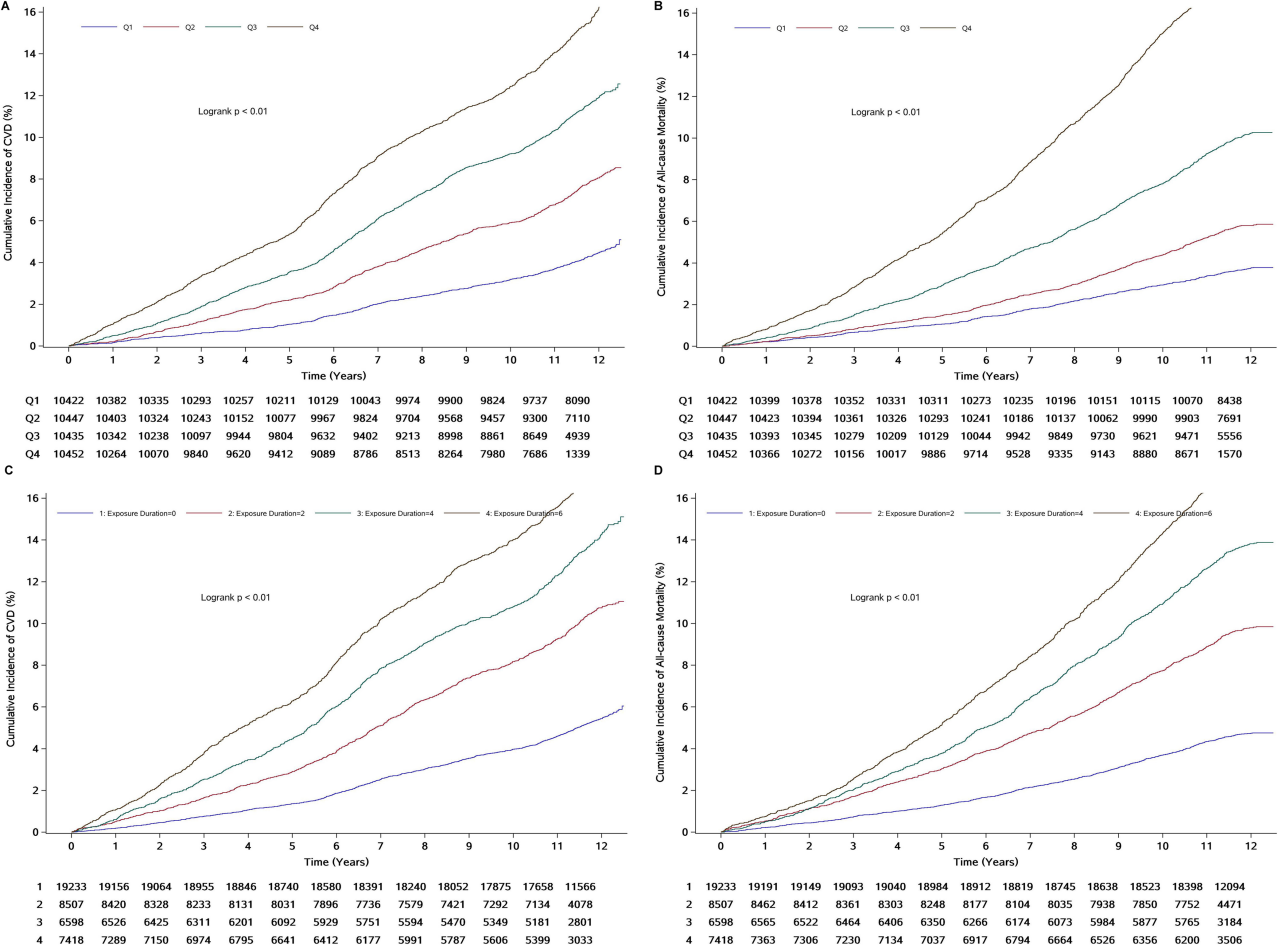
Kaplan–Meier curves of CVD and all‐cause mortality by quartiles of cumulative METS‐VF and exposure duration of high METS‐VF. (A) Cumulative METS‐VF and CVD. (B) Cumulative METS‐VF and all‐cause mortality. (C) Exposure duration of high METS‐VF and CVD. (D) Exposure duration of high METS‐VF and all‐cause mortality. CVD, cardiovascular disease; METS‐VF, metabolic score for visceral fat.

During the median follow‐up of 12.01 years, 4008 (9.60%) CVD events occurred, of which 2808 stroke events, 650 myocardial infarction events, 811 heart failure events and 3944 all‐cause mortality events were observed. Incidence rates per 1000 person‐years of CVD increased significantly from 3.88 (95% CI, 3.54–4.24) in Q1 group to 14.11 (95% CI, 13.41–14.84) in Q4 group; incidence rates per 1000 person‐years of all‐cause mortality increased significantly from 3.13 (95% CI, 2.83–3.45) in Q1 group to 16.70 (95% CI, 15.95–17.46) in Q4 group. Kaplan–Meier curves also showed that the cumulative incidence of CVD and all‐cause mortality increased with increasing cumulative METS‐VF (log‐rank test, *p* < 0.05; Figure 2).

After adjusting for potential covariates, compared to participants in Q1 group, the HRs of incident CVD were 1.55 (95% CI, 1.38–1.74), 2.13 (95% CI, 1.91–2.38), 2.78 (95% CI, 2.49–3.17) for those in Q2 group, in Q3 group and in Q4 group; the HRs of incident all‐cause mortality were 1.59 (95% CI, 1.40–1.81), 2.67 (95% CI 2.37–3.01) and 4.90 (95% CI 4.36–5.50) for those in Q2 group, in Q3 group and in Q4 group (Table 2). With per SD increment in cumulative METS‐VF, HRs for CVD and all‐cause mortality were increased by 44% (95% CI 1.39–1.49) and 77% (95% CI 1.71–1.83), respectively. In addition, there were a non‐linear association between cumulative METS‐VF and risk of CVD and all‐cause mortality (Figure S1).

**TABLE 2:** Association of cumulative exposure to METS‐VF with the risk of cardiovascular disease and all‐cause mortality.

	Cardiovascular disease			All‐cause mortality		
	Case/total	Incidence ratio	HR (95% CI)	Case/total	Incidence ratio	HR (95% CI)
Cumulative exposure						
Q1	478/10422	3.88 (3.54–4.24)	Reference	392/10422	3.13 (2.83–3.45)	Reference
Q2	842/10447	6.99 (6.54–7.48)	1.55 (1.38–1.74)	609/10447	4.90 (4.53–5.31)	1.59 (1.40–1.81)
Q3	1191/10435	10.38 (9.80–10.98)	2.13 (1.91–2.38)	1060/10435	8.82 (8.31–9.37)	2.67 (2.37–3.01)
Q4	1497/10452	14.11 (13.41–14.84)	2.78 (2.49–3.10)	1883/10452	16.70 (15.95–17.46)	4.90 (4.36–5.50)
P for trend	—	—	< 0.01	—	—	< 0.01
Per SD increment	4008/41756	8.63 (8.36–8.90)	1.44 (1.39–1.49)	3944/41756	8.17 (7.92–8.43)	1.77 (1.71–1.83)
Exposure duration						
0 years	1043/19233	4.67 (4.40–4.97)	Reference	907/19233	3.99 (3.73–4.25)	Reference
2 years	870/8507	9.24 (8.65–9.88)	1.77 (1.61–1.95)	829/8507	8.46 (7.91–9.06)	2.24 (2.03–2.47)
4 years	888/6598	12.56 (11.76–13.42)	2.29 (2.08–2.53)	906/6598	12.14 (11.37–12.96)	3.36 (3.04–3.72)
6 years	1207/7418	15.75 (14.89–16.67)	2.70 (2.45–2.97)	1302/7418	15.81 (14.98–16.69)	4.45 (4.02–4.92)
*p* for trend	—	—	< 0.01	—	—	< 0.01

*Note:* Incidence ratio is per 1000 person‐years. All models adjusted for LDL‐C, ALT, hs‐CRP categories, BMI categories, hypertension, diabetes mellitus, dyslipidaemia, drinking status, smoking status, physical exercise and educational status.

Abbreviations: CI, confidence interval; HR, hazard ratio; METS‐VF, metabolic score for visceral fat; Q, quartile; SD, standard deviation.

**FIGURE 3:**
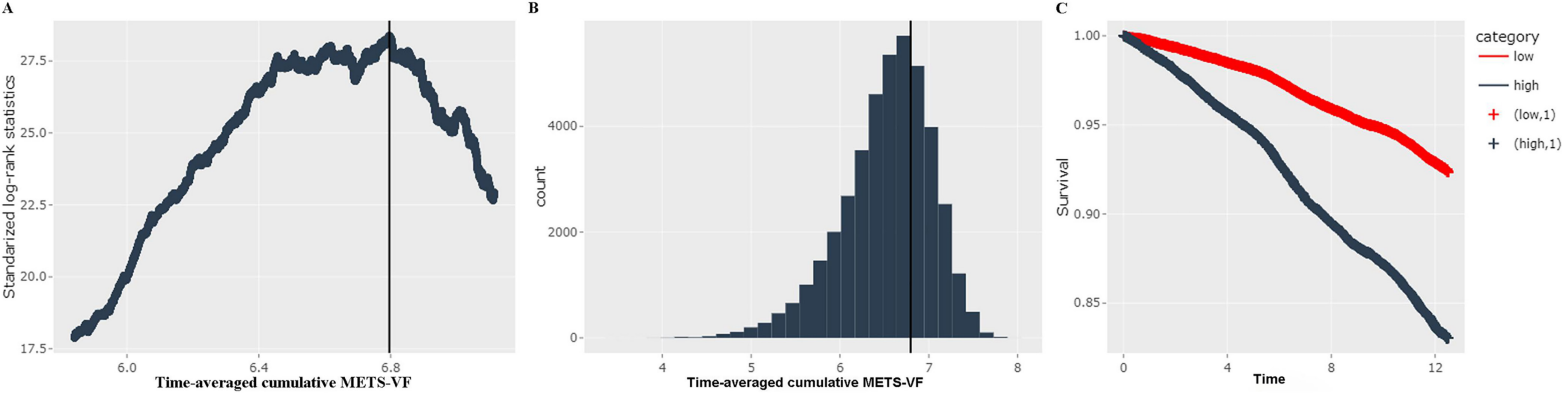
Determining cut‐off values of time‐averaged cumulative METS‐VF with maxstat algorithm. (A) Plot of standardized log‐rank statistics. (B) Histogram of time‐averaged cumulative METS‐VF. (C) Kaplan–Meier plot according to optimized cut‐point of time‐averaged cumulative METS‐VF. METS‐VF, metabolic score for visceral fat.

Using a survival‐time method to calculate maximally selected rank statistics, the optimal cut‐off value of time‐averaged cumulative METS‐VF associated with CVD incident was ≥ 6.80 (Figure 3). HRs for CVD and all‐cause mortality were increased with exposure duration of high METS‐VF increasing. Compared with participants without exposure of high METS‐VF, the HRs of CVD and all‐cause mortality were increased by 77% (95% CI 1.61–1.95) and 114% (95% CI, 2.03–2.47) for those with 2 years exposure of high METS‐VF, 129% (95% CI 2.08–2.53) and 236% (95% CI, 3.04–3.72) for those with 4 years exposure of high METS‐VF, 170% (95% CI 2.45–2.97) and 345 (95% CI, 4.02–4.92) for those with 6 years' exposure of high METS‐VF. The associations of cumulative exposure to METS‐VF with CVD subtypes were still consistent (Tables S2–S4).

### 3.3 ROC Analysis for the Predictive Performances of Cumulative METS‐VF for Cardiovascular Disease and All‐Cause Mortality

**FIGURE 4:**
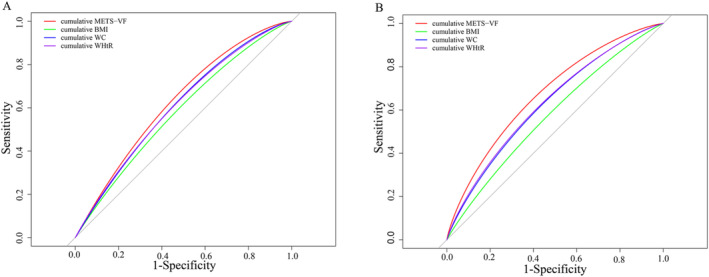
Comparison of predictive performances for cardiovascular disease and all‐cause mortality between cumulative METS‐VF and other obesity indexes (cumulative BMI, cumulative WC and cumulative WHtR). (A) Cardiovascular disease. (B) All‐cause mortality. BMI, body mass index; METS‐VF, metabolic score for visceral fat; WC, waist circumference; WHtR, waist‐to‐height ratio.

The result of ROC curve analysis showed that cumulative METS‐VF had the highest predictive for CVD among 4 indexes including cumulative METS‐VF, cumulative waist circumference, cumulative BMI and cumulative WHtR (Figure 4). The AUC of cumulative METS‐VF was 0.627, whereas the AUCs were 0.606 for cumulative waist circumference, 0.606 for cumulative WHtR and 0.581 for cumulative BMI (Table S5). The Youden index of cumulative METS‐VF (Youden index, 0.191) for CVD was higher than that of waist circumference (Youden index, 0.160), WHtR (Youden index, 0.162) and BMI (Youden index, 0.119). In addition, the cumulative METS‐VF also had the largest AUCs for predicting risk of all‐cause mortality.

### 3.4 Subgroup and Sensitivity Analyses

The multiplicative interactions were found between age, BMI, WHtR, hypertension, diabetes mellitus and dyslipidaemia and cumulative METS‐VF on the risk of CVD (Table 3). We observed similar results for the association with all‐cause mortality, but there was not interaction between age and cumulative METS‐VF, and there was an interaction between hs‐CRP and cumulative METS‐VF. The HRs of CVD for per SD increment in cumulative METS‐VF were 1.29 (95% CI, 1.23–1.36) in participants with an age < 60 years versus 1.18 (95% CI, 1.11–1.25) in participants with an age ≥ 60 years (< 60 years‐to‐ ≥ 60 years ratio of HR: 1.09 [95% CI, 1.01–1.18]), the HRs of CVD were 1.57 (95% CI, 1.48–1.67) in participants with BMI < 24 kg/m^2^ versus 1.38 (95% CI, 1.33–1.44) in participants with BMI ≥ 24 kg/m^2^ (< 24 kg/m^2^‐to‐ ≥ 24 kg/m^2^ ratio of HR: 1.14 [95% CI, 1.06–1.22]), the HRs of CVD were 1.56 (95% CI, 1.44–1.68) in participants with WHtR < 0.5 versus 1.38 (95% CI, 1.32–1.43) in participants with WHtR ≥ 0.5 (< 0.5‐to‐ ≥ 0.5 ratio of HR: 1.13 [95% CI, 1.04–1.23]), the HRs of CVD were 1.60 (95% CI, 1.51–1.70) in participants without hypertension versus 1.35 (95% CI, 1.30–1.41) in participants with hypertension (no‐to‐yes ratio of HR: 1.19 [95% CI, 1.10–1.27]), the HRs of CVD were 1.49 (95% CI, 1.44–1.55) in participants without diabetes mellitus versus 1.22 (95% CI, 1.13–1.32) in participants with diabetes mellitus (no‐to‐yes ratio of HR: 1.22 [95% CI, 1.12–1.33]), and the HRs of CVD were 1.49 (95% CI, 1.43–1.56) in participants without dyslipidaemia versus 1.34 (95% CI, 1.27–1.42) in participants with diabetes dyslipidaemia (no‐to‐yes ratio of HR: 1.11 [95% CI, 1.04–1.19]).

**TABLE 3:** Subgroup analyses for the association of per SD increment in cumulative METS‐VF with the risk of cardiovascular disease and all‐cause mortality.

	Cardiovascular disease			All‐cause mortality		
Subgroup categories	HR (95% CI)	Group 1 to Group 2 ratio of HR (95% CI)	*p* for interaction	HR (95% CI)	Group 1 to Group 2 ratio of HR (95% CI)	*p* for interaction
Age, years						
< 60	1.29 (1.23–1.36)	1.09 (1.01–1.18)	0.02	1.22 (1.15–1.30)	0.94 (0.87–1.02)	0.17
≥ 60	1.18 (1.11–1.25)			1.29 (1.22–1.35)		
Sex						
Females	1.51 (1.37–1.65)	1.09 (0.98–1.20)	0.10	1.73 (1.56–1.92)	1.02 (0.92–1.14)	0.83
Males	1.39 (1.34–1.44)			1.69 (1.63–1.75)		
BMI, kg/m^2^						
< 24	1.57 (1.48–1.67)	1.14 (1.06–1.22)	< 0.01	1.87 (1.77–1.97)	1.10 (1.03–1.18)	< 0.01
≥ 24	1.38 (1.33–1.44)			1.70 (1.63–1.77)		
WHtR						
< 0.5	1.56 (1.44–1.68)	1.13 (1.04–1.23)	< 0.01	1.89 (1.76–2.03)	1.11 (1.02–1.20)	0.02
≥ 0.5	1.38 (1.32–1.43)			1.71 (1.65–1.78)		
Hypertension						
No	1.60 (1.51–1.70)	1.19 (1.10–1.27)	< 0.01	1.91 (1.81–2.02)	1.14 (1.06–1.21)	< 0.01
Yes	1.35 (1.30–1.41)			1.68 (1.61–1.75)		
Diabetes mellitus						
No	1.49 (1.44–1.55)	1.22 (1.12–1.33)	< 0.01	1.84 (1.78–1.91)	1.24 (1.14–1.35)	< 0.01
Yes	1.22 (1.13–1.32)			1.48 (1.37–1.59)		
Dyslipidaemia						
No	1.49 (1.43–1.56)	1.11 (1.04–1.19)	< 0.01	1.84 (1.77–1.91)	1.14 (1.07–1.23)	< 0.01
Yes	1.34 (1.27–1.42)			1.61 (1.52–1.71)		
Hs‐CRP, mg/dL						
< 3	1.43 (1.38–1.49)	0.99 (0.91–1.06)	0.72	1.70 (1.63–1.77)	0.89 (0.83–0.96)	< 0.01
≥ 3	1.45 (1.35–1.54)			1.91 (1.79–2.02)		

*Note:* Group 1 includes participants who are age<60 years, females, BMI < 24 kg/m^2^, WHtR < 0.5, without hypertension, without diabetes mellitus, without dyslipidaemia or hs‐CRP < 3 mg/dL; Group 2 includes participants who are age ≥ 60 years, males, BMI ≥ 24 kg/m^2^, WHtR ≥ 0.5, hypertension, diabetes mellitus, dyslipidaemia or hs‐CRP ≥ 3 mg/dL. All models adjusted for LDL‐C, ALT, hs‐CRP categories, BMI categories, hypertension, diabetes mellitus, dyslipidaemia, drinking status, smoking status, physical exercise and educational status.

Abbreviations: BMI, body mass index; CI, confidence interval; HR, hazard ratio; Hs‐CRP, high‐sensitive C‐reactive protein; METS‐VF, metabolic score for visceral fat; SD, standard deviation; WHtR, waist‐to‐height ratio.

In several sensitivity analyses, the associations of cumulative METS‐VF with CVD and all‐cause mortality were not altered by the exclusion of participants who had incident CVD within 2 years; or by exclusion of participants with eGFR lower than 60 mL/min per 1.73 m^2^; or by exclusion of participants with use of antihypertensive medications, hypoglycaemic medications or lipid‐lowering medications; or by exclusion of participants with history of cancer; or considering death as a competing risk (Tables S6 and S7).

## 4 Discussion

The primary finding of the current study is that the high cumulative METS‐VF was associated with increased risks of CVD and all‐cause mortality. Furthermore, the risks for CVD and all‐cause mortality significantly increased with exposure duration of the high METS‐VF and were more significant at a normal BMI level.

The METS‐VF is a novel surrogate indicator calculated based on a non‐insulin index (METS‐IR), WHtR, age and sex and has been shown to assess visceral fat; in addition, the indicator can apply to assess cardio‐metabolic risk in clinical and epidemiological research [7]. Growing evidence suggests that obesity is a leading risk factor associated with CVD. Inflammation in white adipose tissues (especially visceral fat) plays an essential role in the development of CVD and is implicated in the pathogenesis of CVD. BMI is a commonly used indicator of obesity that represents general obesity or waist circumference; however, BMI cannot distinguish body fat distribution or quantify visceral fat mass. For example, regardless of BMI, Asians have higher visceral fat accumulation compared to their Caucasian counterparts [24]. A study by Rush, Freitas, and Plank revealed that Asian Indians have a higher body fat percentage than other Asians and Caucasians for the same BMI [25]. Furthermore, Filipino women, despite having similar waist circumference measurements, exhibited higher levels of visceral fat and visceral‐to‐subcutaneous abdominal fat ratios compared to their Caucasian counterparts [26]. In particular, previous studies have demonstrated that the METS‐IR can predict fat regions, indicating that METS‐VF has better performance and simplicity than traditional measures [7, 16]. Several studies have convincingly demonstrated the advantages of METS‐VF in predicting the risk of chronic kidney disease, hypertension and diabetes mellitus [27, 28, 29]. In a retrospective study including 6827 participants, the METS‐VF at baseline was independently associated with the risk of CVD and all‐cause mortality, comprising individuals with normal glucose tolerance [8]. Nonetheless, the study is limited by a small sample size, no detailed subgroup of CVD and a single measurement of METS‐VF.

To our knowledge, it is the first prospective cohort study with a long follow‐up to investigate the association between METS‐VF exposure and the risk of CVD and all‐cause mortality. This study integrated exposure intensity and exposure duration and found that increasing exposure duration and cumulative exposure of METS‐VF were significantly associated with increased risk for CVD and its subtypes (stroke, myocardial infarction and heart failure). In addition, METS‐VF, as a novel visceral indicator, was associated with an increased risk of all‐cause mortality in the general population. Our results have confirmed that longitudinal changes in METS‐VF play an essential role in CVD development. A review study investigating the effect of obesity duration on cardiometabolic disease risk also supports the findings of our study [30]. In addition, the optimal cut‐off value for time‐averaged cumulative METS‐VF associated with CVD incidence was determined, revealing a dramatic increase in the risk of CVD and mortality as the levels of cumulative METS‐VF rose above 6.80. Overall, METS‐VF represents a cost‐effective, simple, convenient and reliable indicator of visceral fat and may help identify individuals at risk of CVD. Determining the optimal cut‐off value will facilitate the application of this indicator. In addition to age and sex, all components of METS‐VF are modifiable risk factors, highlighting the importance of lifestyle modification and medication intervention in limiting disease progression.

The present study also identified interactions between METS‐VF and BMI levels, hypertension status and diabetes status. The results revealed that the increase in cumulative exposure to METS‐VF was more strongly associated with CVD in non‐obese individuals with a BMI < 24 kg/m^2^ compared to obese individuals. This indirectly supports the notion that the association between obesity and CVD may be related to the distribution of fat rather than the overall degree of obesity. Additionally, the future risk of developing CVD increases with prolonged exposure to high METS‐VF. Previous studies indicated that an increase in cumulative exposure to CVAI (another reliable surrogate marker for assessing fat distribution and function) is associated with a higher risk of CVD, which is consistent with our findings [31]. Furthermore, the predictive model of our study also supports the conclusion that the accumulation of visceral fat has a higher predictive value compared to traditional obesity assessment indicators. The findings also suggested that the association between visceral fat accumulation and the risk of CVD was more pronounced in individuals without hypertension or diabetes, which may be related to hypertension and diabetes. Relevant research has found that the population attributable fraction of CVD due to elevated systolic blood pressure and increased blood glucose levels is significantly higher than that due to BMI [32]. This also suggests that the accumulation of visceral fat should not be overlooked in individuals with hypertension while focusing on controlling blood pressure. In contrast, the threat of visceral fat accumulation may be underestimated in individuals without hypertension due to their generally healthier status.

## 5 Limitations

Nevertheless, the limitations of the present study should be acknowledged. First, this study is an observational study; although many confounding factors were adjusted for, including biochemical markers and lifestyle factors, this study cannot prove causality. In addition, other uncontrolled confounding factors might have an impact on the association, and further studies are required to validate our findings. Second, the visceral fat was assessed using a recognized formula in this study, which still cannot reflect the detailed distribution of visceral fat. METS‐VF is an indirect indicator for assessing visceral fat, which provides a practical evaluation method for daily life and clinical practice. Third, the overall study population included more males than females in the Kailuan Study, but the sample size of the female population was still large enough to perform the analyses. Finally, confounders cannot be completely ruled out due to the limitations of the initial study design. Still, the limitations were mitigated as much as possible by adjusting for multiple covariates, ensuring a large sample size and complete follow‐up pathways.

### 5.1 Future Directions

Future directions may include further exploration of the following areas. Firstly, the mechanisms underlying the accumulation of visceral obesity and the increased risk of CVD should be explored. Secondly, the higher risk of CVD in individuals with normal body weight but with visceral fat accumulation compared to obese individuals requires additional investigation. Finally, studies are needed to examine whether improvements in visceral fat levels can reduce the future risk of CVD.

## 6 Conclusions

In conclusion, our study confirmed that high cumulative METS‐VF was associated with an increased risk of CVD and all‐cause mortality. This association was stronger as exposure to high METS‐VF was prolonged. The association of METS‐VF with the risk of CVD was stronger in the young to middle populations and populations with normal BMI. The cumulative METS‐VF is superior to other obesity indexes in predicting the risk of CVD and mortality. Early striving to control the long‐time METS‐VF and control at the target levels of METS‐VF could be of enormous benefit for CVD risk reduction. Notably, treatment should be individualized based on health status during health management.

## Ethics Statement

The study was performed according to the guidelines of the Helsinki Declaration and was approved by the Ethics Committee of Kailuan General Hospital (approval number: 2006‐05). All participants were agreed to take part in the study and signed an informed written consent.

## Consent

The authors have nothing to report.

## Conflicts of Interest

The authors declare no conflicts of interest.

## Supporting information


**Figure S1** HR and 95% CI for the association of cumulative METS‐VF with the risk of CVD and all‐cause mortality by using restricted cubic spline regression with 3 knots with placed at the 25th, 50th and 75th percentiles. (A) Cumulative METS‐VF and the risk of CVD. (B) Cumulative METS‐VF and the risk of all‐cause mortality. The red lines indicate HR, the blue lines indicate 95% CI. CVD, cardiovascular disease; CI, confidence interval; HR, hazard ratio; METS‐VF, metabolic score for visceral fat.
**Table S1.** Baseline characteristics of study participants included and excluded.
**Table S2.** Association of cumulative exposure to METS‐VF with the risk of stroke.
**Table S3**. Association of cumulative exposure to METS‐VF with the risk of myocardial infarction.
**Table S4.** Association of cumulative exposure to METS‐VF with the risk of heart failure.
**Table S5.** Predictive performances of cumulative METS‐VF, cumulative BMI, cumulative WC and cumulative WHtR for cardiovascular disease and all‐cause mortality.
**Table S6.** Sensitivity analysis for the association of cumulative METS‐VF with the risk of cardiovascular disease.
**Table S7.** Sensitivity analysis for the association of cumulative METS‐VF with the risk of all‐cause mortality.

## Data Availability

The datasets used and/or analysed during the current study are available from the corresponding author on reasonable request.
