# Supplementary material for: Association of Cumulative Exposure to Metabolic Score for Visceral Fat With the Risk of Cardiovascular Disease and All‐Cause Mortality: A Prospective Cohort Study
Source: J Cachexia Sarcopenia Muscle. 2025 Feb 11;16(1):e13702. doi: 10.1002/jcsm.13702 (PMC11814533; doi:10.1002/jcsm.13702)
Supplement: Supplementary file 1 — Figure S1 HR and 95% CI for the association of cumulative METS‐VF with the risk of CVD and all‐cause mortality by using restricted cubic spline regression with 3 knots with placed at the 25th, 50th and 75th percentiles. (A) Cumulative METS‐VF and the risk of CVD. (B) Cumulative METS‐VF and the risk of all‐cause mortality. The red lines indicate HR, the blue lines indicate 95% CI. CVD, cardiovascular disease; CI, confidence interval; HR, hazard ratio; METS‐VF, metabolic score for visceral fat. Table S1. Baseline characteristics of study participants included and excluded. Table S2. Association of cumulative exposure to METS‐VF with the risk of stroke. Table S3. Association of cumulative exposure to METS‐VF with the risk of myocardial infarction. Table S4. Association of cumulative exposure to METS‐VF with the risk of heart failure. Table S5. Predictive performances of cumulative METS‐VF, cumulative BMI, cumulative WC and cumulative WHtR for cardiovascular disease and all‐cause mortality. Table S6. Sensitivity analysis for the association of cumulative METS‐VF with the risk of cardiovascular disease. Table S7. Sensitivity analysis for the association of cumulative METS‐VF with the risk of all‐cause mortality. [file JCSM-16-e13702-s001.docx]

**Supplement contents**

**Figure S1 HR and 95%CI for the association of cumulative METS-VF with the risk of CVD and all-cause mortality by using restricted cubic spline regression with 3 knots with placed at the 25th, 50th, and 75th percentiles**

A: Cumulative METS-VF and the risk of CVD; B: Cumulative METS-VF and the risk of all-cause mortality. The red lines indicate HR, the blue lines indicate 95%CI. CVD, cardiovascular disease; METS-VF, metabolic score for visceral fat; HR, hazard ratio; CI, confidence interval.

**Table S1 Baseline characteristics of study participants included and excluded**

**Table S2 Association of cumulative exposure to METS-VF with the risk of stroke**

**Table S3 Association of cumulative exposure to METS-VF with the risk of myocardial infarction**

**Table S4 Association of cumulative exposure to METS-VF with the risk of heart failure**

**Table S5 Predictive performances of cumulative METS-VF, cumulative BMI, cumulative WC, and cumulative WHtR for cardiovascular disease and all-cause mortality**

**Table S6 Sensitivity analysis for the association of cumulative METS-VF with the risk of cardiovascular disease**

**Table S7 Sensitivity analysis for the association of cumulative METS-VF with the risk of all-cause mortality**

**Code for performing the statistical analysis**
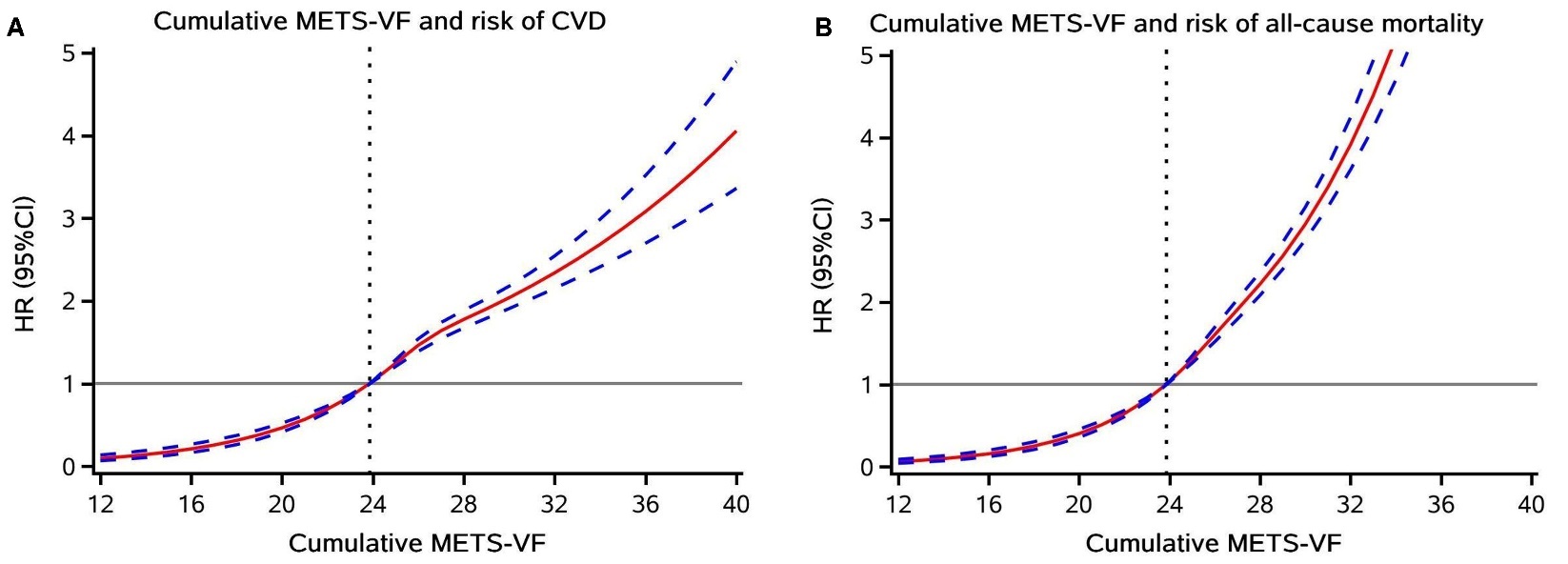


**Figure S1 HR and 95%CI for the association of cumulative METS-VF with the risk of CVD and all-cause mortality by using restricted cubic spline regression with 3 knots with placed at the 25th, 50th, and 75th percentiles**

A: Cumulative METS-VF and the risk of CVD; B: Cumulative METS-VF and the risk of all-cause mortality. The red lines indicate HR, the blue lines indicate 95%CI. CVD, cardiovascular disease; METS-VF, metabolic score for visceral fat; HR, hazard ratio; CI, confidence interval.

**Table S1 Baseline characteristics of study participants included and excluded**

| Characteristics | Included | Excluded | P value |
| --- | --- | --- | --- |
| Number of participants | 41756 | 59754 |  |
| Age, years | 48.70±11.50 | 54.19±12.96 | <0.01 |
| Male, n (%) | 32792 (78.53) | 48318 (80.86) | <0.01 |
| BMI, kg/m^2^ | 25.23±3.31 | 24.91±3.60 | <0.01 |
| WHtR | 0.52±0.06 | 0.52±0.06 | 0.23 |
| CVAI | 91.73±42.08 | 97.16±44.89 | <0.01 |
| LDL-C, mmol/L | 2.34±0.91 | 2.37±0.89 | <0.01 |
| ALT, IU/L | 18.00 (13.00, 25.00) | 18.00 (12.00, 24.00) | <0.01 |
| Hs-CRP, mg/dL | 0.73 (0.28, 2.10) | 0.90 (0.33, 2.30) | <0.01 |
| eGFR, mL/min/1.73m^2^ | 84.56±24.91 | 80.11±26.01 | <0.01 |
| Current smokers, n (%) | 14131 (34.72) | 19664 (34.14) | 0.06 |
| Current drinkers, n (%) | 15740 (38.66) | 20912 (36.30) | <0.01 |
| Active physical activity, n (%) | 5609 (13.43) | 9742 (17.18) | <0.01 |
| High school or above, n (%) | 8047 (19.27) | 12427 (21.17) | <0.01 |
| Hypertension, n (%) | 16935 (40.56) | 27798 (47.06) | <0.01 |
| Diabetes mellitus, n (%) | 3418 (8.19) | 6197 (10.59) | <0.01 |
| Dyslipidemia, n (%) | 13491 (32.31) | 19934 (33.36) | <0.01 |
| Antihypertensive medications, n (%) | 3339 (8.00) | 7984 (13.36) | <0.01 |
| Hypoglycemic medications, n (%) | 736 (1.76) | 1745 (2.92) | <0.01 |
| Lipid-lowering medications, n (%) | 279 (0.67) | 684 (1.14) | <0.01 |

Abbreviation: METS-VF, metabolic score for visceral fat; BMI, body mass index; WHtR, waist-to-height ratio; LDL-C, low-density lipoprotein cholesterol; ALT, alanine aminotransferase; Hs-CRP, high sensitive C-reactive protein; eGFR, estimated glomerular filtration rate.

**Table S2 Association of cumulative exposure to METS-VF with the risk of stroke**

|  | Case/Total | Incidence ratio | HR (95%CI) |
| --- | --- | --- | --- |
| **Cumulative exposure** | | | |
| Q1 | 361/10422 | 2.91 (2.63-3.23) | Reference |
| Q2 | 614/10447 | 5.05 (4.67-5.46) | 1.50 (1.32-1.72) |
| Q3 | 842/10435 | 7.22 (6.75-7.72) | 2.03 (1.79-2.31) |
| Q4 | 991/10452 | 9.12 (8.57-9.71) | 2.51 (2.21-2.86) |
| P for trend | - | - | <0.01 |
| Per SD increment | 2808/41756 | 5.96 (5.75-6.19) | 1.39 (1.34-1.45) |
| **Exposure Duration** | | | |
| 0 years | 766/19233 | 3.41 (3.18-3.66) | Reference |
| 2 years | 635/8507 | 6.66 (6.16-7.20) | 1.77 (1.59-1.97) |
| 4 years | 608/6598 | 8.44 (7.79-9.14) | 2.16 (1.92-2.42) |
| 6 years | 799/7418 | 10.13 (9.45-10.86) | 2.45 (2.18-2.75) |
| P for trend | - | - | <0.01 |

Abbreviation: METS-VF, metabolic score for visceral fat; Q, quartile; SD, standard deviation; HR, hazard ratio; CI, confidence interval. Incidence ratio is per 1000 person-years. All models adjusted for LDL-C, ALT, hs-CRP categories, BMI categories, hypertension, diabetes mellitus, dyslipidemia, drinking status, smoking status, physical exercise, and educational status.

**Table S3 Association of cumulative exposure to METS-VF with the risk of myocardial infarction**

|  | Case/Total | Incidence ratio | HR (95%CI) |
| --- | --- | --- | --- |
| **Cumulative exposure** | | | |
| Q1 | 75/10422 | 0.60 (0.48-0.75) | Reference |
| Q2 | 132/10447 | 1.07 (0.90-1.27) | 1.43 (1.07-1.90) |
| Q3 | 214/10435 | 1.80 (0.57-2.06) | 2.14 (1.63-2.82) |
| Q4 | 229/10452 | 2.05 (1.08-2.33) | 2.28 (1.72-3.01) |
| P for trend | - | - | <0.01 |
| Per SD increment | 650/41756 | 1.36 (1.26-1.46) | 1.33 (1.22-1.45) |
| **Exposure Duration** | | | |
| 0 years | 160/19233 | 0.71 (0.60-0.82) | Reference |
| 2 years | 141/8507 | 1.45 (1.23-1.71) | 1.72 (1.36-2.18) |
| 4 years | 144/6598 | 1.95 (1.65-1.29) | 2.10 (1.64-2.69) |
| 6 years | 205/7417 | 2.52 (2.20-2.89) | 2.48 (1.95-3.16) |
| P for trend | - | - | <0.01 |

Abbreviation: METS-VF, metabolic score for visceral fat; Q, quartile; SD, standard deviation; HR, hazard ratio; CI, confidence interval. Incidence ratio is per 1000 person-years. All models adjusted for LDL-C, ALT, hs-CRP categories, BMI categories, hypertension, diabetes mellitus, dyslipidemia, drinking status, smoking status, physical exercise, and educational status.

**Table S4 Association of cumulative exposure to METS-VF with the risk of heart failure**

|  | Case/Total | Incidence ratio | HR (95%CI) |
| --- | --- | --- | --- |
| **Cumulative exposure** | | | |
| Q1 | 62/10422 | 0.50 (0.39-0.64) | Reference |
| Q2 | 138/10447 | 1.12 (0.95-1.32) | 1.98 (1.46-2.68) |
| Q3 | 218/10435 | 1.83 (1.60-2.09) | 2.82 (2.11-3.77) |
| Q4 | 393/10452 | 3.54 (3.21-3.91) | 4.87 (3.67-6.46) |
| P for trend | - | - | <0.01 |
| Per SD increment | 811/41756 | 1.70 (1.58-1.82) | 1.69 (1.57-1.82) |
| **Exposure Duration** | | | |
| 0 years | 163/19233 | 0.72 (0.62-0.84) | Reference |
| 2 years | 149/8507 | 1.53 (1.31-1.80) | 1.93 (1.53-2.2) |
| 4 years | 194/6598 | 2.63 (2.29-3.03) | 3.13 (2.49-3.93) |
| 6 years | 305/7418 | 3.78 (3.37-4.22) | 4.15 (3.32-5.19) |
| P for trend | - | - | <0.01 |

Abbreviation: METS-VF, metabolic score for visceral fat; Q, quartile; SD, standard deviation; HR, hazard ratio; CI, confidence interval. Incidence ratio is per 1000 person-years. All models adjusted for LDL-C, ALT, hs-CRP categories, BMI categories, hypertension, diabetes mellitus, dyslipidemia, drinking status, smoking status, physical exercise, and educational status.

**Table S5 Predictive performances of cumulative METS-VF, cumulative BMI, cumulative WC, and cumulative WHtR for cardiovascular disease and all-cause mortality**

|  | AUC (95%CI) | Cut-off | Sensitivity and specificity, % | Youden index | P for comparison |
| --- | --- | --- | --- | --- | --- |
| **Cardiovascular disease** | | | | | |
| Cumulative METS-VF | 0.627 (0.618-0.635) | 26.37 | 0.620/0.571 | 0.191 | Reference |
| Cumulative WC | 0.606 (0.597-0.614) | 341.91 | 0.695/0.465 | 0.160 | <0.001 |
| Cumulative WHtR | 0.606 (0.598-0.615) | 1.31 | 0.640/0.522 | 0.162 | <0.001 |
| Cumulative BMI | 0.581 (0.572-0.590) | 64.98 | 0.564/0.555 | 0.119 | <0.001 |
| **All-cause mortality** | | | | | |
| Cumulative METS-VF | 0.683 (0.674-0.691) | 27.02 | 0.644/0.649 | 0.293 | Reference |
| Cumulative WC | 0.634 (0.626-0.643) | 223.10 | 0.617/0.590 | 0.207 | <0.001 |
| Cumulative WHtR | 0.640 (0.631-0.649) | 1.31 | 0.665/0.552 | 0.217 | <0.001 |
| Cumulative BMI | 0.578 (0.569-0.587) | 64.82 | 0.511/0.607 | 0.118 | <0.001 |

Abbreviation: METS-VF, metabolic score for visceral fat; BMI, body mass index; WC, waist circumference; WHtR, waist-to-height ratio; AUC, area under the curve; CI, confidence interval.

**Table S6 Sensitivity analysis for the association of cumulative METS-VF with the risk of cardiovascular disease**

|  | Quartiles of cumulative METS-VF | | | | P for trend |
| --- | --- | --- | --- | --- | --- |
|  | Q1 | Q2 | Q3 | Q4 |  |
| **Sensitivity analysis 1** | | | | | |
| Case/Total | 435/10379 | 770/10375 | 1079/10323 | 1280/10235 | - |
| HR (95%CI) | Reference | 1.57 (1.40-1.77) | 2.18 (1.94-2.45) | 2.73 (2.43-3.07) | <0.01 |
| **Sensitivity analysis 2** | | | | | |
| Case/Total | 439/9913 | 797/9894 | 1076/9645 | 1300/9454 | - |
| HR (95%CI) | Reference | 1.59 (1.41-1.79) | 2.14 (1.91-2.41) | 2.75 (2.45-3.09) | <0.01 |
| **Sensitivity analysis 3** | | | | | |
| Case/Total | 371/9412 | 582/8561 | 819/8131 | 1018/7903 | - |
| HR (95%CI) | Reference | 1.56 (1.37-1.78) | 2.29 (2.02-2.60) | 3.04 (2.67-3.45) | <0.01 |
| **Sensitivity analysis 4** | | | | | |
| Case/Total | 476/10378 | 839/10400 | 1179/10364 | 1488/10345 | - |
| HR (95%CI) | Reference | 1.55 (1.39-1.74) | 2.13 (1.90-2.38) | 2.78 (2.49-3.11) | <0.01 |
| **Sensitivity analysis 5** | | | | | |
| Case/Total | 478/10422 | 842/10447 | 1191/10435 | 1497/10452 | - |
| HR (95%CI) | Reference | 1.55 (1.38-1.74) | 2.13 (1.91-2.38) | 2.78 (2.49-3.10) | <0.01 |

Abbreviation: METS-VF, metabolic score for visceral fat; HR, hazard ratio; CI, confidence interval; Q, quartile. All models adjusted for LDL-C, ALT, hs-CRP categories, BMI categories, hypertension, diabetes mellitus, dyslipidemia, drinking status, smoking status, physical exercise, and educational status.

Sensitivity analysis 1: exclusion of participants with incident cardiovascular disease within 2 years;

Sensitivity analysis 2: exclusion of participants with eGFR lower than 60 mL/min per 1.73 m^2^;

Sensitivity analysis 3: exclusion of participants with antihypertensive medications, hypoglycemic medications, or lipid-lowering medications;

Sensitivity analysis 4: exclusion of participants with history of cancer;

Sensitivity analysis 5: sensitivity analysis used competing risk model, considering death as a competing risk.

**Table S7 Sensitivity analysis for the association of cumulative METS-VF with the risk of all-cause mortality**

|  | Quartiles of cumulative METS-VF | | | | P for trend |
| --- | --- | --- | --- | --- | --- |
|  | Q1 | Q2 | Q3 | Q4 |  |
| **Sensitivity analysis 1** | | | | | |
| Case/Total | 383/10379 | 586/10375 | 1021/10323 | 1787/10235 | - |
| HR (95%CI) | Reference | 1.58 (1.39-1.80) | 2.67 (2.37-3.02) | 4.90 (4.36-5.51) | <0.01 |
| **Sensitivity analysis 2** | | | | | |
| Case/Total | 349/9913 | 547/9894 | 865/9645 | 1515/9454 | - |
| HR (95%CI) | Reference | 1.61 (1.41-1.85) | 2.55 (2.24-2.90) | 4.75 (4.20-5.38) | <0.01 |
| **Sensitivity analysis 3** | | | | | |
| Case/Total | 329/9412 | 465/8561 | 762/8131 | 1341/7903 | - |
| HR (95%CI) | Reference | 1.65 (1.43-1.91) | 2.77 (2.42-3.16) | 5.24 (4.61-5.96) | <0.01 |
| **Sensitivity analysis 4** | | | | | |
| Case/Total | 378/10378 | 595/10400 | 1035/10364 | 1831/10345 | - |
| HR (95%CI) | Reference | 1.61 (1.42-1.84) | 2.70 (2.39-3.05) | 4.94 (4.39-5.56) | <0.01 |

Abbreviation: METS-VF, metabolic score for visceral fat; HR, hazard ratio; CI, confidence interval; Q, quartile. All models adjusted for LDL-C, ALT, hs-CRP categories, BMI categories, hypertension, diabetes mellitus, dyslipidemia, drinking status, smoking status, physical exercise, and educational status.

Sensitivity analysis 1: exclusion of participants with incident cardiovascular disease within 2 years;

Sensitivity analysis 2: exclusion of participants with eGFR lower than 60 mL/min per 1.73 m^2^;

Sensitivity analysis 3: exclusion of participants with antihypertensive medications, hypoglycemic medications, or lipid-lowering medications;

Sensitivity analysis 4: exclusion of participants with history of cancer.

**Code** **for performing the statistical analysis**

**Code in SAS software:**

title 'the association of cumulative METS-VF with risk of CVD';

proc phreg data=c2;

class zu bmi_zu smoke10 drink10 phy hyp10 tnb10 /param=ref ref=first ;

model suifang_cvd*cvd(0)=zu ldl_10 alt_10 bmi_zu crp smoke10 drink10 phy hyp10 tnb10 dys edu_zu/rl;

run;

proc phreg data=c2;

class bmi_zu smoke10 drink10 phy hyp10 tnb10 /param=ref ref=first ;

model suifang_cvd*cvd(0)=t_mets_vf_sd ldl_10 alt_10 bmi_zu crp smoke10 drink10 phy hyp10 tnb10 dys edu_zu/rl;

run;

proc phreg data=c2;

class zu_dur bmi_zu smoke10 drink10 phy hyp10 tnb10 /param=ref ref=first ;

model suifang_cvd*cvd(0)=zu_dur ldl_10 alt_10 bmi_zu crp smoke10 drink10 phy hyp10 tnb10 dys edu_zu/rl;

run;

title 'the association of cumulative METS-VF with risk of all-cause mortality ';

proc phreg data=c2;

class zu bmi_zu smoke10 drink10 phy hyp10 tnb10 /param=ref ref=first ;

model suifang_death*death(0)=zu ldl_10 alt_10 bmi_zu crp smoke10 drink10 phy hyp10 tnb10 dys edu_zu/rl;

run;

proc phreg data=c2;

class bmi_zu smoke10 drink10 phy hyp10 tnb10 /param=ref ref=first ;

model suifang_death*death(0)=t_mets_vf_sd ldl_10 alt_10 bmi_zu crp smoke10 drink10 phy hyp10 tnb10 dys edu_zu/rl;

run;

proc phreg data=c2;

class zu_dur bmi_zu smoke10 drink10 phy hyp10 tnb10 /param=ref ref=first ;

model suifang_death*death(0)=zu_dur ldl_10 alt_10 bmi_zu crp smoke10 drink10 phy hyp10 tnb10 dys edu_zu/rl;

run;

title "incidence ratio";

proc summary data=c2 nway;

var suifang_cvd cvd;

class zu;

output out=rates(drop=_type_ _freq_) sum=suifang_cvd cvd;

run;

data rates;

set rates;

_rate=1000*(cvd/suifang_cvd);

ci_low=_rate/exp(1.96*sqrt(1/cvd));

ci_high=_rate*exp(1.96*sqrt(1/cvd));

run;

proc print data=rates;run;

**Scripts used in the R software:**

list.of.packages <- c("shiny","survival", "OptimalCutpoints", "scales", "survminer", "rolr", "pROC", "datasets", "survMisc", "maxstat", "heatmaply", "shinythemes", "htmlwidgets", "ggplot2", "plotly", "GGally", "d3heatmap")

new.packages <- list.of.packages[!(list.of.packages %in% installed.packages()[,"Package"])]

if(length(new.packages)) install.packages(new.packages)

lapply(list.of.packages,function(x){library(x,character.only=TRUE)})
